# Midlife Lifestyle Activities Moderate APOE ε4 Effect on *in vivo* Alzheimer’s Disease Pathologies

**DOI:** 10.3389/fnagi.2020.00042

**Published:** 2020-02-27

**Authors:** So Yeon Jeon, Min Soo Byun, Dahyun Yi, Jun-Ho Lee, Kang Ko, Bo Kyung Sohn, Jun-Young Lee, Seung-Ho Ryu, Dong Woo Lee, Seoung A Shin, Yu Kyeong Kim, Koung Mi Kang, Chul-Ho Sohn, Dong Young Lee

**Affiliations:** ^1^Department of Psychiatry, Chungnam National University Hospital, Daejeon, South Korea; ^2^Department of Neuropsychiatry, Seoul National University Bundang Hospital, Seongnam, South Korea; ^3^Institute of Human Behavioral Medicine, Medical Research Center, Seoul National University, Seoul, South Korea; ^4^Department of Psychiatry, National Center for Mental Health, Seoul, South Korea; ^5^Department of Neuropsychiatry, Sanggye Paik Hospital, Inje University College of Medicine, Seoul, South Korea; ^6^Department of Neuropsychiatry, SMG-SNU Boramae Medical Center, Seoul, South Korea; ^7^Department of Psychiatry, School of Medicine, Konkuk University Medical Center, Konkuk University, Seoul, South Korea; ^8^Department of Nuclear Medicine, SMG-SNU Boramae Medical Center, Seoul, South Korea; ^9^Department of Radiology, Seoul National University Hospital, Seoul, South Korea; ^10^Department of Psychiatry, Seoul National University College of Medicine, Seoul, South Korea

**Keywords:** Alzheimer’s disease, APOE ε4, *in vivo* pathology, midlife, physical activity, cognitive activity

## Abstract

This study aimed to investigate whether the midlife cognitive activity and physical activity moderate the relationship between apolipoprotein Eε4 (APOE4) and *in vivo* Alzheimer’s disease (AD) pathologies. In total, 287 non-demented older adults (mean age 72 years) from the Korean Brain Aging Study for the Early diagnosis and prediction of Alzheimer’s disease cohort were included. Participants underwent a comprehensive clinical assessment including the evaluation for midlife CA and physical activity, [^11^C]-Pittsburgh-Compound-B-positron emission tomography (PET), [^18^F]-fluorodeoxyglucose PET, structural magnetic resonance imaging (MRI), and APOE genotyping. We used linear regression and regression-based mediated-moderation models for statistical analyses. Neither midlife cognitive activity nor physical activity moderated the effect of APOE4 on β-amyloid (Aβ) retention itself. Midlife cognitive activity significantly moderated the effect of APOE4 on hippocampal volume [*B* (SE) = − 627.580 (252.327), *t* = −2.488, *p* = 0.014]: APOE4 carriers had smaller hippocampal volume than non-carriers at relatively high cognitive activity state (*p* = 0.004), but not at relatively low cognitive activity condition (*p* = 0.937). Midlife physical activity significantly moderated the effect of Aβ retention, which was closely related to APOE4, on AD-signature region cerebral glucose metabolism [AD-CM; *B* (SE) = 0.004 (0.002), *t* = 2.030, *p* = 0.043]: higher Aβ accumulation was associated with lower AD-CM in relatively low physical activity condition (*p* < 0.001), whereas no such association was observed in relatively high physical activity state (*p* = 0.791). The findings suggest that high midlife cognitive activity may accelerate hippocampal atrophy induced by APOE4, whereas high midlife physical activity may delay AD-related cerebral hypometabolism by weakening the influence of APOE4-associated Aβ retention.

## Background

The apolipoprotein ε4 (APOE4) is the most well-evidenced risk gene for Alzheimer’s disease (AD; Corder et al., 1993) and is related to *in vivo* AD pathologies such as β-amyloid (Aβ) accumulation (Morris et al., 2010), reduced hippocampal volume (Hashimoto et al., 2001), and decreased AD-signature region cerebral glucose metabolism (AD-CM; Small et al., 1995; Lowe et al., 2014). APOE4 has complex effects on AD pathophysiology through both Aβ-mediated pathway (i.e., indirect effect of APOE4 on hippocampal volume or AD-CM reduction *via* Aβ accumulation) and Aβ-independent pathways (i.e., direct effect of APOE4 on hippocampal volume or AD-CM reduction not mediated by Aβ accumulation; Huang, 2010).

While APOE4 is a non-modifiable genetic risk factor, modifiable factors such as cognitive activity and physical activity have been associated with a decreased risk of cognitive decline (Ngandu et al., 2015) and AD dementia (Rovio et al., 2005; Kivipelto et al., 2008; Najar et al., 2019). However, studies on the *in vivo* neuropathological mechanisms underlying the association between cognitive activity or physical activity and AD-related cognitive decline have produced controversial findings (Valenzuela et al., 2008; Erickson et al., 2009; Liang et al., 2010; Bugg and Head, 2011; Head et al., 2012; Landau et al., 2012; Vemuri et al., 2012, 2016, 2017; Brown et al., 2013b; Wirth et al., 2014; Gidicsin et al., 2015; Ko et al., 2018). Such modifiable lifestyle activities may change the AD pathophysiological processes associated with APOE4. However, their moderation for the influence of APOE4 on AD pathologies remains poorly understood (Kivipelto et al., 2008; Head et al., 2012; Wirth et al., 2014).

Some previous studies have adopted current cognitive activity or physical activity to investigate the relationship between lifestyle activities and *in vivo* AD pathologies (Valenzuela et al., 2008; Erickson et al., 2009; Landau et al., 2012; Brown et al., 2013b; Wirth et al., 2014). However, as AD pathology, Aβ deposition, in particular, precedes the clinical symptom onset of dementia by 10–15 years (Villemagne et al., 2013), current activity itself could be affected by pre-existing AD pathology (i.e., reverse causation; de Bruijn et al., 2013; Jack et al., 2013b). In contrast, midlife cognitive and physical activities are less likely to be affected by AD pathology. Moreover, many previous studies indicated that such midlife activities are related with a decreased risk of late-life cognitive decline (Karp et al., 2009; Inzelberg et al., 2013; Najar et al., 2019) and AD dementia (Rovio et al., 2005; Andel et al., 2008; Kivipelto et al., 2008; Tolppanen et al., 2015).

Therefore, we aimed to investigate whether the midlife cognitive activity and physical activity can moderate the effect of APOE4 on *in vivo* AD pathologies measured by neuroimaging modalities.

## Materials and Methods

### Participants

The present study included 287 non-demented older adults [215 cognitively normal (CN), 72 mild cognitive impairment (MCI)] between 55 and 90 years of age who participated in the Korean Brain Aging Study for the Early Diagnosis and Prediction of Alzheimer’s disease (KBASE), an ongoing prospective cohort study initiated in 2014 (Byun et al., 2017). The CN group consisted of participants with a Clinical Dementia Rating (CDR; Morris, 1993) score of 0. All individuals with MCI met the core clinical criteria for MCI diagnosis recommended by the National Institute of Aging and Alzheimer’s Association guidelines (Albert et al., 2011), which are as follows: (1) memory complaints confirmed by an informant; (2) objective memory impairments; (3) preserved global cognitive function; (4) independance in functional activities; and (5) no dementia. Regarding Criterion 2, the age-, education-, and sex-adjusted z-scores for at least one of four episodic memory tests were < −1.0. The four memory tests were the Word List Memory, Word List Recall, Word List Recognition, and Constructional Recall tests, which are included in the Korean version of the Consortium to Establish a Registry for Alzheimer’s Disease (CERAD-K) neuropsychological battery (Lee et al., 2004). All MCI individuals had a CDR score of 0.5. The exclusion criteria were as follows: (1) presence of a major psychiatric illness, including alcohol-related disorders; (2) significant neurological or medical conditions or comorbidities that could affect mental function; (3) contraindications for an magnetic resonance imaging (MRI) scan (e.g., pacemaker or claustrophobia); (4) illiteracy; (5) the presence of significant visual/hearing difficulties and/or severe communication or behavioral problems that would make clinical examinations or brain scans difficult; (6) taking an investigational drug; and (7) pregnant or breastfeeding. All the participants received comprehensive neuropsychological and clinical evaluation including midlife cognitive activity and physical activity according to the KBASE assessment protocol (Byun et al., 2017). More detailed information on the KBASE study methodology including the enrollment and assessment of participants was described previously (Byun et al., 2017).

The Institutional Review Board of Seoul National University Hospital (C-1401-027-547) and Seoul Metropolitan Government-Seoul National University Boramae Medical center (26-2015-60) in South Korea approved the present study and all volunteers provided written informed consent prior to participation.

### APOE Genotyping

Blood samples were obtained *via* venipuncture and DNA was extracted from whole blood. APOE genotyping was performed as described in a previous study (Park et al., 2017). If an individual has at least one APOE4 allele, we defined it as an APOE4 carrier.

### Assessment of Midlife Cognitive and Physical Activities

#### Cognitive Activity

The cognitive activity of each subject was assessed using a 39 item expanded version (Wilson et al., 2005) of a previously reported 25-item autobiographical self-report questionnaire (Wilson et al., 2003; Landau et al., 2012). This questionnaire has sufficient internal consistency and temporal stability (Wilson et al., 2003, 2005). Participants were asked to report how often they engaged in common cognitively demanding activities with few barriers to participation, such as reading newspapers, magazines, or books; visiting a museum or library; attending a concert, play or musical and writing letters or a diary, at 5 age epochs: 6, 12, 18, and 40 years and the current age. Responses for each item were made using a 5-point frequency scale: 5, every day or almost every day; 4, several times a week; 3, several times a month; 2, several times a year; and 1, once a year or less. Among the 39 items, nine items were for current age (i.e., late-life) cognitive activity and nine items are for midlife (40 years of age) cognitive activity. The item scores for current age and midlife were averaged to yield current- and midlife cognitive activity value, respectively.

#### Physical Activity

Midlife physical activity (age 40–55 years) was assessed using the interviewer-administered Lifetime Total Physical Activity Questionnaire, a tool with demonstrated reliability (Friedenreich et al., 1998, 2004) and validity (Gill et al., 2015). This questionnaire assesses occupational, household, and leisure activities separately throughout a respondent’s lifetime. The frequency and duration of these activities were assessed by recording the number of years, months per year, weeks per month, days per week and hours per day that each activity was performed. The intensity of activity was estimated by the participant as sedentary, light, moderate or heavy. A metabolic equivalent (MET) value was assigned to each activity based on the *Compendium of Physical Activities* (Ainsworth et al., 2011). The index of midlife- and current physical activity was the average MET-hr./week spent on leisure activity at the ages of between 40–55 years old and over the past 3 years each. We selected leisure activities, but not occupational or household activities because we wanted to include only a modifiable factor that could be controlled. Most previous studies about the influence of physical activity on AD or dementia risk have focused only on leisure-time physical activity (Rovio et al., 2005; Tolppanen et al., 2015; Krell-Roesch et al., 2018).

### Assessment of AD Neuroimaging Biomarkers

#### Measurement of Cerebral Aβ Accumulation

All subjects underwent simultaneous three-dimensional (3D) PiB-PET and T1-weighted MRI using a 3.0 T Biograph mMR (PET-MR) scanner (Siemens, Washington, DC, USA) according to the manufacturer’s approved guidelines. After 40 min from intravenous administration of 555 MBq of 11C-PiB (range, 450–610 MBq), the PiB-PET image data were collected in list mode (5 min × 6 frames). All PiB-PET images were processed with routine corrections for uniformity, UTE-based attenuation, and decay corrections, and reconstructed into a 256 × 256 image matrix using iterative methods (six iterations with 21 subsets). T1-weighted 3D MR images were acquired in the sagittal orientation with the following parameters; repetition time = 1,670 ms, echo time = 1.89 ms, field of view 250 mm, 256 × 256 matrix with 1.0 mm slice thickness. The image preprocessing was performed using Statistical Parametric Mapping 12 (Wellcome Department of Cognitive Neurology, London, UK^1^) and Individual Brain Atlases using Statistical Parametric Mapping software (IBASPM^2^). First, static PiB-PET images were co-registered to individual T1-weighted MR images and then transformation parameters for spatial normalization of individual T1-weighted MR images to a standard Montreal Neurological Institute (MNI) template were calculated. The inverse transformation parameters were used to transform coordinates from the automatic anatomic labeling (AAL) 116 atlas (Tzourio-Mazoyer et al., 2002) into an individual space for each subject (a resampling voxel size = 1 × 0.98 × 0.98 mm), and the non-gray matter portions of the atlas were individually masked using the cerebral gray matter segment image from each subject. Cerebellar gray matter was used as the reference region and mean [^11^C]-PiB uptake value was extracted from all the cerebellar lobular regions except for the vermis from a probabilistic cerebellar atlas (Institute of Cognitive Neuroscience, UCL; Cognitive Neuroscience Laboratory, Royal Holloway).

The AAL algorithm (Tzourio-Mazoyer et al., 2002) and a region combining method (Reiman et al., 2009) were applied to determine regions of interest (ROI) to characterize the [^11^C]-PiB level in the frontal, lateral parietal, posterior cingulate-precuneus, and lateral temporal regions. The standardized uptake value ratio (SUVR) values for each ROI were calculated by dividing the mean value for all voxels within each ROI by the mean cerebellar gray matter uptake value in the same image. A global cortical ROI consisting of the 4 ROIs was also defined, and a global Aβ retention value was calculated by dividing the mean PiB uptake value for all voxels of the global cortical ROIs by the mean cerebellar gray matter uptake value (Reiman et al., 2009). The global Aβ retention values had skewed distribution and were log-transformed in the analysis.

#### Hippocampal Volume Measurement

All T1-weighted images were acquired in the sagittal orientation using the abovementioned 3.0 T PET-MR machine. All MR images were automatically segmented using FreeSurfer version 5.3^3^ with manual correction of minor segmentation errors. An adjusted hippocampal volume (HVa) was calculated as the unstandardized residual from the linear regression of total hippocampal volume (HVt) vs. the total intracranial volume (ICV) of the reference group (the young CN group of the study cohort; Lee et al., 2017). HVa indicates the volume deviated from the expected HVt according to the ICV in young CN subjects.

#### Measurement of AD-Signature Cerebral Glucose Metabolism

All subjects also underwent FDG-PET imaging using the same PET-MR machine as described previously. The participants fasted for at least 6 h and rested in a waiting room for 40 min prior to the scans after intravenous administration of 0.1 mCi/Kg of [^18^F]-FDG radioligands. The PET data collected in list mode (5 min × 4 frames) were processed for routine corrections such as uniformity, UTE-based attenuation, and decay corrections. After inspecting the data for any significant head movements, we reconstructed them into a 20-min summed image using iterative methods (6 iterations with 21 subsets). The following image processing steps were performed using SPM12^4^ implemented in Matlab 2014a (Mathworks, Natick, MA, USA). First, static FDG-PET images were co-registered to individual T1 structural images, and transformation parameters for the spatial normalization of individual T1 images to a standard MNI template were calculated and used to spatially normalize the PET images to the MNI template. After smoothing the spatially normalized FDG-PET images with a 12-mm Gaussian filter, intensity normalization was performed using the pons as the reference region. AD-signature FDG ROIs, such as the angular gyri, posterior cingulate cortex, and inferior temporal gyri, which are sensitive to the changes associated with AD (Jack et al., 2014) were determined. AD-CM was defined as a voxel-weighted mean SUVR extracted from the AD-signature FDG ROIs.

### Statistical Analysis

**Figure 1:**
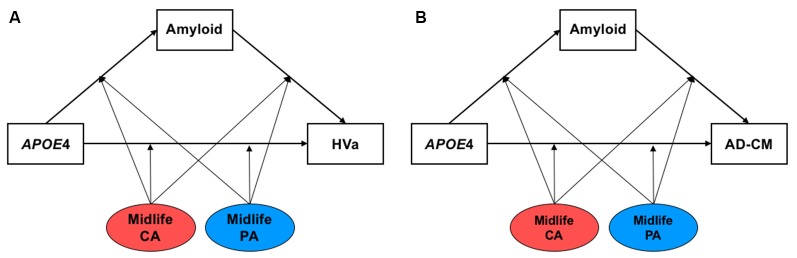
The hypothetical moderated mediation model to analyze the associations of apolipoprotein E ε4 (APOE4) with β-amyloid (Aβ) retention and **(A)** HVa or **(B)** AD-CM, and the moderation effect of cognitive and physical activity on the associations. The sequence of Alzheimer’s disease (AD) pathologies are based on hypothetical amyloid cascade model of AD pathophysiology (Jack et al., 2010; Jack et al., 2013a). APOE4, APOE ε4; CA, cognitive activity; PA, physical activity; HVa, adjusted hippocampal volume; AD-CM, AD-signature region cerebral glucose metabolism.

First, multiple linear regression analyses were conducted to examine the simple associations between midlife cognitive activity (or physical activity) and AD biomarkers using IBM SPSS Statistics software 23 (IBM Corp., Armonk, NY, USA). Then, we tested models including both Aβ-mediated- and Aβ-independent pathways of APOE4 effects using the Process Macro program (Hayes, 2017) to investigate systematically the effects of APOE4 on AD biomarkers and moderation by midlife cognitive activity or physical activity (Figure 1). The inference was determined by 95% bias-corrected bootstrap confidence intervals from 10,000 bootstrap samples. An effect was considered significant if the 95% confidence interval did not include zero. For all the analyses, age, sex, educational year, and clinical diagnosis (CN vs. MCI) were controlled as covariates, and *p*-value < 0.05 was considered significant.

### Data Availability

The datasets generated and analyzed during the present study are not publicly available, owing to ethical considerations and privacy restrictions. Data may be obtained from the corresponding author after approval by the Institutional Review Board of the Seoul National University Hospital, South Korea has been sought.

## Results

### Participant Characteristics

The characteristics of the subjects are shown in Table 1. Of the 287 study participants, 66 (23.0%) were APOE4 carriers. There were no differences between APOE4 carriers and non-carriers regarding age, sex, education, or midlife cognitive activity or physical activity. The proportion of MCI subjects was higher in the APOE4 carrier than in the non-carrier group. APOE4 carriers also had higher global Aβ accumulation, smaller HVa, and lower AD-CM than those of non-carriers.

**Table 1:** Patient characteristics in the overall sample and in the strata by apolipoprotein E ε4 (APOE4) status (*N* = 287).

	All participants (*N* = 287)	APOE4 non-carrier (*n* = 221)	APOE4 carrier (*n* = 66)	*p*-value^a^
**Demographics**
Age (year)	71.91 ± 6.64	71.50 ± 6.83	73.23 ± 5.83	0.064
Sex (F %)	158 (55.05%)	120 (54.30%)	38 (57.58%)	0.742
Educational year (year)	11.19 ± 4.81	11.36 ± 4.92	10.70 ± 4.44	0.329
MCI, no. (%)	72 (25.09%)	44 (19.91%)	28 (42.42%)	<0.001
**Lifestyle enrichment variables**
Midlife physical activity (MET-hr/week)	17.04 ± 33.00	18.48 ± 36.05	12.35 ± 19.48	0.074
Midlife cognitive activity, score	2.30 ± 0.80	2.31 ± 0.81	2.26 ± 0.78	0.630
**AD biomarkers**
Global β-amyloid burden (SUVR)^b^	0.24 ± 0.24	0.18 ± 0.20	0.41 ± 0.29	<0.001
HVa (mm^3^)	−1,143.81 ± 1,011.63	−1,006.85 ± 936.01	−1,621.28 ± 1,123.20	<0.001
AD-CM (SUVR)	1.38 ± 0.13	1.39 ± 0.12	1.34 ± 0.14	0.004

*Values are n (%) or mean (SD). ^a^Comparison between APOE4 carriers and non-carriers by *t*-test for continuous variables and chi-square test for categorical variables. ^b^Coded as ln(global β-amyloid burden). APOE4, apolipoprotein ɛ4; MCI, mild cognitive impairment; SUVR, standardized uptake value ratio; HVa, adjusted hippocampal volume; AD-CM, AD-signature region cerebral glucose metabolism*.

### Simple Associations of APOE4, Cognitive Activity and Physical Activity With AD Biomarkers

Linear regression analyses showed that APOE4 positivity was significantly associated with increased global Aβ retention, decreased HVa, and decreased AD-CM (Table 2). In contrast, neither midlife cognitive activity nor physical activity was related to any of the AD neuroimaging biomarkers.

**Table 2:** Association of APOE4 and midlife activities with AD biomarkers (*N* = 287).

	Amyloid^a^					HVa					AD-CM				
	*B*	*SE*	*β*	*t*	*p*^b^	*B*	*SE*	*β*	*t*	*p*^c^	*B*	*SE*	*β*	*t*	*p*^c^
**APOE4**	0.175	0.029	0.303	6.099	<0.001	−340.045	113.776	−0.141	−2.989	0.003	−0.049	0.019	−0.148	−2.567	0.011
**CA**	0.022	0.020	0.072	1.080	0.281	93.335	77.388	0.075	1.206	0.229	0.006	0.013	0.036	0.468	0.640
**PA**	0.001	0.001	−0.020	−0.389	0.698	0.151	1.581	0.005	0.095	0.924	0.001	0.001	0.044	0.737	0.462

*^a^Coded as ln(global β-amyloid burden). ^b^By multiple linear regression analysis controlling for age, sex, educational year and clinical diagnosis as covariates. ^c^By multiple linear regression analysis controlling for age, sex, educational year, APOE4 status and clinical diagnosis as covariates. HVa, adjusted hippocampal volume; AD-CM, AD-signature region cerebral glucose metabolism; APOE4, apolipoprotein ɛ4; CA, cognitive activity; PA, physical activity*.

### Moderation of Midlife Lifestyle Activities for the Association of APOE4 With Aβ Retention and HVa

**Figure 2:**
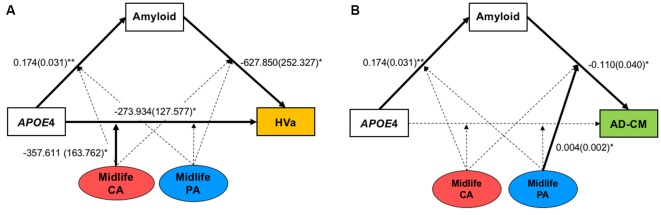
Results from moderated mediation model analyses for the associations of APOE4 with Aβ retention and **(A)** HVa or **(B)** AD-CM, and the moderation effect of midlife cognitive and physical activity for the associations. Values are standardized regression coefficients (standard error) for the associations or moderation effect with statistical significance. Bold lines also indicate significant association or moderation effect. *p < 0.05, **p < 0.005. APOE4, apolipoprotein ε4; CA, cognitive activity; PA, physical activity; HVa, adjusted hippocampal volume; AD-CM, AD-signature region cerebral glucose metabolism.

**Figure 3:**
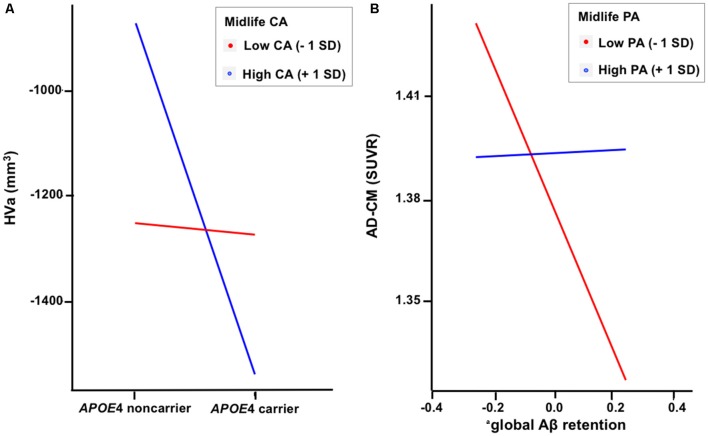
Plots to demonstrate the moderation effect of **(A)** midlife cognitive activity on the relationship between APOE4 and HVa and **(B)** midlife physical activity on the relationship between Aβ retention and AD-CM. ^a^Coded as ln(global β-amyloid burden). APOE4, apolipoprotein ε4; CA, cognitive activity; PA, physical activity; HVa, adjusted hippocampal volume; AD-CM, AD-signature region cerebral glucose metabolism; <1 SD, 1 standard deviation below mean value; +1 SD,: 1 SD above mean value.

When a model including the moderating effect of midlife cognitive and physical activity for the association between APOE4, Aβ retention, and HVa (Figure 1A) was analyzed, midlife cognitive activity significantly moderated the Aβ-independent effect of APOE4 on HVa (Table 3 and Figure 2A). For the purpose of demonstration, the association between APOE4 carrier status and HVa was plotted for each of the high and low midlife cognitive activity state (Figure 3A). At relatively high cognitive activity (1SD above mean) condition, APOE4 carriers had significantly smaller HVa than non-carriers [*B* (SE) = −561.576 (193.159), *t* = −2.907, *p* = 0.004], whereas no such difference was found APOE4 carriers and non-carriers at relatively low cognitive activity (1 SD below mean) condition [*B* (SE) = 13.707 (173.037), *t* = 0.079, *p* = 0.937] (Figure 3A). In contrast, midlife cognitive activity moderated neither the APOE4 effect on Aβ retention itself nor the Aβ-mediated effect of APOE4 on HVa (Table 3 and Figure 2A). In contrast to midlife cognitive activity, midlife physical activity did not show any moderating effect on the influence of APOE4 on Aβ retention and HVa (Table 3 and Figure 2A). Even after current cognitive and physical activity were controlled in the model as additional covariates, the results were unchanged.

**Table 3:** Moderation of midlife activities for the association of APOE4 with Aβ retention and HVa: results from moderated mediation analysis based on PROCESS (*N* = 287).

	Amyloid^a^				HVa			
	*B*	*SE*	*t*	*p*^ b^	*B*	*SE*	*t*	*p*^b^
APOE4	0.174	0.031	5.656	<0.001	−273.934	127.577	−2.147	0.033
CA	0.024	0.020	1.168	0.244	112.472	77.641	1.449	0.149
PA	−0.001	0.001	−0.561	0.575	−1.107	1.941	−0.570	0.569
APOE4 × CA	0.030	0.038	0.798	0.425	−357.611	163.762	−2.184	0.030
APOE4 × PA	0.001	0.002	0.402	0.688	−0.857	6.813	−0.126	0.900
Amyloid^a^					−627.850	252.327	−2.488	0.014
Amyloid^a^ × CA					177.416	272.268	0.652	0.515
Amyloid^a^ × PA					−3.862	13.225	−0.292	0.771

*^a^Coded as ln(global β-amyloid burden). ^b^Adjusted for age, sex, educational year and clinical diagnosis. HVa, adjusted hippocampal volume; APOE4, apolipoprotein e4; CA, cognitive activity; PA, physical activity*.

### Moderation of Midlife Lifestyle Activities for the Association of APOE4 With Aβ Retention and AD-CM

While midlife cognitive activity did not have any moderation effect for the association between APOE4, Aβ retention, and AD-CM, midlife physical activity significantly moderated the effect of Aβ retention on AD-CM (Table 4 and Figure 2B), suggesting the effect of global Aβ retention, which is closely related to APOE4, on AD-CM can be changed by midlife physical activity level. For the purpose of demonstration, the association between global Aβ retention and AD-CM was plotted for each of the high and low midlife physical activity state (Figure 3B). At relatively low physical activity (1SD below mean) condition, higher global Aβ retention was significantly associated with lower AD-CM [*B* (SE) = −16.205 (0.179), *t* = −4.271, *p* < 0.001], whereas no such association was observed in relatively high physical activity (1 SD above mean) condition [*B* (SE) = 0.023(0.089), *t* = 0.264, *p* = 0.791] (Figure 3B). In contrast, midlife physical activity moderated neither the effect of APOE4 on Aβ retention nor the Aβ-independent effect of APOE4 on AD-CM (Table 4 and Figure 3B). Even after current cognitive and physical activity were controlled in the model as additional covariates, the results were similar.

**Table 4:** Moderation of midlife activities for the association of APOE4 with Aβ retention and AD-CM: results from moderated mediation analysis based on PROCESS (*N* = 287).

	Amyloid^a^				AD-CM			
	*B*	*SE*	*t*	*p*^b^	*B*	*SE*	*t*	*p*^b^
APOE4	0.174	0.031	5.656	<0.001	−0.022	0.020	−1.090	0.277
CA	0.024	0.020	1.168	0.244	0.010	0.012	0.811	0.418
PA	−0.001	0.001	−0.561	0.575	0.001	0.001	0.850	0.396
APOE4 × CA	0.030	0.038	0.798	0.425	−0.042	0.026	−1.623	0.106
APOE4 × PA	0.001	0.002	0.402	0.688	−0.001	0.001	−1.257	0.210
Amyloid^a^					−0.110	0.040	−2.738	0.007
Amyloid^a^ CA					−0.021	0.043	−0.476	0.635
Amyloid^a^ PA					0.004	0.002	2.030	0.043

*^a^Coded as ln(global β-amyloid burden). ^b^Adjusted for age, sex, educational year and clinical diagnosis. AD-CM, AD-signature region cerebral glucose metabolism; APOE4, apolipoprotein ɛ4; CA, cognitive activity; PA, physical activity*.

## Discussion

We observed that APOE4 was strongly associated with increased global Aβ accumulation and reduced HVa and AD-CM, whereas neither midlife cognitive activity nor physical activity was related to any of the AD biomarkers in bivariate association analysis. In terms of the moderating effects of midlife lifestyle activities, midlife cognitive activity moderated the Aβ-independent influence of APOE4 on HVa, and midlife physical activity moderated the Aβ-mediated influence of APOE4 on AD-CM, while neither activity moderated the APOE4 effects on Aβ accumulation.

### Association Between APOE4, CA, and PA With AD Biomarkers

Consistent with previous reports (Small et al., 1995; Hashimoto et al., 2001; Morris et al., 2010; Lowe et al., 2014), APOE4 status was strongly associated with Aβ accumulation and the neurodegeneration biomarkers in our study.

In contrast, neither midlife cognitive activity nor physical activity was related to any of the AD neuroimaging biomarkers in bivariate analysis. Many studies investigating the association between cognitive activity or physical activity and AD biomarkers have reported inconsistent findings (Valenzuela et al., 2008; Erickson et al., 2009; Liang et al., 2010; Bugg and Head, 2011; Head et al., 2012; Landau et al., 2012; Vemuri et al., 2012, 2016, 2017; Brown et al., 2013b; Wirth et al., 2014; Gidicsin et al., 2015; Ko et al., 2018). Among them, only a few have focused on the effect of midlife activities (Vemuri et al., 2016, 2017; Ko et al., 2018) and have shown no direct association between midlife cognitive activity or physical activity and AD biomarkers, which was similar to our findings. Such a null association with Aβ accumulation and AD-related neurodegeneration biomarkers appears discordant with the finding that midlife lifestyle activities are associated with a decreased risk of late-life cognitive decline (Ngandu et al., 2015) or AD dementia (Rovio et al., 2005; Najar et al., 2019). Such a discrepancy was also observed in prior studies (Wilson et al., 2013; Gidicsin et al., 2015). A report based on the Harvard Aging Brain Study demonstrated that a history of greater cognitive activity is correlated with better cognitive performance, but not with Aβ accumulation, glucose metabolism, or hippocampal volume in CN older adults (Gidicsin et al., 2015). A neuropathological study also showed that greater past cognitive activity is related to slower late-life cognitive decline, independently of AD neuropathologies (Wilson et al., 2013). Taken together, a change in AD pathology itself is not likely to be the direct substrate underlying the effect of past lifestyle activities on cognitive benefit.

### Moderation of Midlife Cognitive Activity or Physical Activity for APOE4 Effects on Aβ Deposition

In our study, neither midlife cognitive activity nor physical activity moderated the APOE4 effect on Aβ deposition itself. However, two previous studies reported a significant interaction effect between lifestyle activities and APOE4 on Aβ accumulation (Head et al., 2012; Wirth et al., 2014). They showed a beneficial effect of cognitive activity (Wirth et al., 2014) or physical activity (Head et al., 2012) on Aβ accumulation only in APOE4 carriers. This discrepancy might be attributed to different study methods and sample characteristics. We focused specifically on midlife activities to reduce the possibility of reverse causation (Jack et al., 2013b), whereas other studies adopted lifetime or recent 10-year lifestyle activities including current ones, which could be affected by underlying pathophysiological processes. In addition, the educational levels of their subjects (mean educational years: 16.86 (Wirth et al., 2014) and 16.23 (Head et al., 2012) were higher than those of our study (11.19 years). Another study (Vemuri et al., 2016) detected an inverse association between midlife cognitive activity and Aβ accumulation in APOE4 carriers only in the high education group (≥14 years), but not in the low education group (<14 years).

### Moderation of Midlife Cognitive Activity for APOE4 Effects on HVa

Midlife cognitive activity moderated the Aβ-independent influence of APOE4 on HVa. More specifically, the direct negative effect of APOE4 on HVa was more evident in individuals with higher midlife cognitive activity than in those with a lower midlife cognitive activity. According to the APOE4 antagonistic pleiotropy hypothesis, APOE4 differentially impacts across different life stages. APOE4 offers cognitive benefits during early adulthood at the expense of a more rapid decline in cognitive function with aging (Tuminello and Han, 2011). Young CN individuals with APOE4 have elevated resting-state activity in the default mode network including the hippocampus compared to those without APOE4 (Filippini et al., 2009). APOE4 carriers in midlife have more strongly activated memory-related brain regions including the hippocampus to maintain the same level of performance than non-carriers, but this neural compensatory recruitment begins to decline by midlife (Bondi et al., 2005; Tuminello and Han, 2011). Such increased activity in the memory-related brain regions is also known to be related to atrophy of the medial temporal lobe including the hippocampus (O’Brien et al., 2010). Taken together, our results indicate that excessive midlife cognitive activity in APOE4 carriers may accelerate hippocampal atrophy by imposing hyperactivation of the related brain regions.

### Moderation of Midlife Physical Activity for APOE4 Effects on AD-CM

While midlife physical activity did not moderate the Aβ-independent influence of APOE4 on AD-CM, it moderated the indirect pathway from APOE4 to AD-CM *via* Aβ accumulation. More specifically, Aβ accumulation, which is closely linked to APOE4, was associated with decreased AD-CM in individuals with a lower level of midlife physical activity, whereas such an inverse correlation between Aβ accumulation and AD-CM was not significant in those with a higher level of physical activity.

There are several possible pathways linking active physical activity and preserved AD-CM. First, physical activity has been suggested to increase cognitive or brain reserve through angiogenesis, increased cerebral blood flow and enhanced synaptic plasticity (van Praag, 2009; Brown et al., 2013a). Individuals with a greater reserve through active physical activity can tolerate a greater burden of cerebral Aβ accumulation and do not show reduced AD-CM as shown in the inactive physical activity group. Second, Aβ shares a consensus amino acid sequence with insulin and Aβ directly binds to the insulin receptor leading to increased insulin-resistance (Xie et al., 2002). Increased insulin resistance is associated with reduced AD-CM (Willette et al., 2015). As active physical activity decreases insulin-resistance (Balkau et al., 2008), it could prevent the reduction of AD-CM by Aβ. Finally, active physical activity also lowers chronic inflammation (Brown et al., 2013a). Increased cerebral Aβ accumulation induces neuroinflammation which can reduce AD-CM (Akiyama et al., 2000). Consistent with our result, a 21-year longitudinal follow-up study reported that midlife physical activity is inversely associated with a late-life risk of AD dementia only among APOE4 carriers (Rovio et al., 2005). Given the strong association between APOE4 and Aβ accumulation (Morris et al., 2010), our findings suggest that higher midlife physical activity decreases APOE4-related AD risk by weakening the influence of Aβ accumulation on further hypometabolism or neurodegeneration.

### Strengths and Limitations

One of the key strengths of this study is the statistical approach using models including both the Aβ-mediated pathway and the Aβ-independent pathway of APOE4 influence. This approach made it possible to clarify the complex associations and interactions between APOE, midlife lifestyle factors, and AD biomarkers. Our findings, based on such complex models, may explain why studies about the effects of lifestyle activities on AD biomarkers have resulted in inconsistent findings. The relatively large sample size, particularly of the CN group, is another strong point of this study. Nevertheless, some limitations should also be mentioned. Because this was not a longitudinal study, we could not confirm causality for the observed associations. To overcome such a limitation in the study design, we used midlife cognitive activity and physical activity instead of current activities. Nevertheless, further long-term follow up studies are needed to clarify the causal aspects. Although the cognitive activity and physical activity questionnaires used in the present study were reliable and well-validated, they are based on self-reports and might be biased due to recall problems. To minimize such recall bias, we only included non-demented subjects. Although MCI individuals have some memory problems, their problems are confined to recent memory, not remote memory (Leyhe et al., 2009).

## Conclusion

The current study was the first attempt to elucidate the moderating effect of modifiable midlife lifestyle factors on the influence of APOE4 on *in vivo* AD pathologies. Our findings suggest that high midlife cognitive activity may accelerate hippocampal atrophy induced by APOE4. In contrast, active midlife physical activity may delay AD-signature regional brain hypometabolism by weakening the influence of APOE4-associated Aβ accumulation. Overall, the information obtained in this study will be helpful to select preventive midlife lifestyle activities to reduce the negative influence of the genetic risk for AD.

## Data Availability Statement

The datasets generated for this study are available on request to the corresponding author.

## Ethics Statement

The studies involving human participants were reviewed and approved by Seoul National University Hospital, Seoul Metropolitan Government-Seoul National University Boramae Medical center. The patients/participants provided their written informed consent to participate in this study.

## Author Contributions

SJ and DYL is responsible for the study concept and design, acquisition, analysis and interpretation of data; and drafting and critically revising the manuscript for intellectual content. MB, DY, J-HL, KK, BS, and J-YL are responsible for the acquisition, analysis, and interpretation of data. S-HR, DWL, SS, YK, KMK, and C-HS participated in the analysis and interpretation of data. All authors read and approved the final manuscript.

## Conflict of Interest

The authors declare that the research was conducted in the absence of any commercial or financial relationships that could be construed as a potential conflict of interest.

## Footnotes

^1^http://www.fil.ion.ucl.ac.uk/spm

^2^http://www.thomaskoenig.ch/Lester/ibaspm.htm

^3^http://surfer.nmr.mgh.harvard.edu/

^4^http://www.fil.ion.ucl.ac.uk/spm

## Abbreviations

APOE4: apolipoprotein ε4 allele
AD: Alzheimer’s disease
Aβ: β-amyloid
AD-CM: AD-signature region cerebral glucose metabolism
CA: cognitive activity
PA: physical activity
CN: cognitively normal
MCI: mild cognitive impairment
KBASE: the Korean Brain Aging Study for the Early Diagnosis and Prediction of AD
CDR: clinical dementia rating
CERAD-K: the Korean version of the Consortium to Establish a Registry for Alzheimer’s Disease Assessment Packet
PiB: [^11^C] Pittsburg compound B
PET: positron emission tomography
FDG: [^18^F]-fluorodeoxyglucose
MET: metabolic equivalent
MNI: Montreal Neurological Institute
AAL: automatic anatomic labeling
ROI: region of interest
SUVR: standardized uptake value ratio
HVa: adjusted hippocampal volume
HVt: total hippocampal volume
ICV: intracranial volume.
